# Integrative Data Augmentation with U-Net Segmentation Masks Improves Detection of Lymph Node Metastases in Breast Cancer Patients

**DOI:** 10.3390/cancers12102934

**Published:** 2020-10-12

**Authors:** Yong Won Jin, Shuo Jia, Ahmed Bilal Ashraf, Pingzhao Hu

**Affiliations:** 1 Department of Biochemistry and Medical Genetics, University of Manitoba, Winnipeg, MB R3E 0J9, Canada; jiny2@myumanitoba.ca (Y.W.J.); jias@myumanitoba.ca (S.J.); 2 Department of Electrical and Computer Engineering, University of Manitoba, Winnipeg, MB R3T 5V6, Canada; ahmed.ashraf@umanitoba.ca; 3 Research Institute in Oncology and Hematology, CancerCare Manitoba, Winnipeg, MB R3E 0V9, Canada

**Keywords:** digital histopathology, computer-assisted diagnosis, deep learning, breast cancer, lymph node metastasis

## Abstract

**Simple Summary:**

In recent years many successful models have been developed to perform various tasks in digital histopathology, yet, there is still a reluctance to fully embrace the new technologies in clinical settings. One of the reasons for this is that although these models have achieved high performance at the patch-level, their performance at the image-level can still be underwhelming. Through this study, our main objective was to investigate whether integrating multiple extracted histological features to the input image had potential to further improve the performance of classifier models at the patch-level. Ideally, by achieving 100% accuracy at the patch-level, one can achieve 100% accuracy at the image-level. We hope that our research will entice the community to develop new strategies to further improve performance of existing state-of-the-art models, and facilitate their adoption in the clinics.

**Abstract:**

Deep learning models have potential to improve performance of automated computer-assisted diagnosis tools in digital histopathology and reduce subjectivity. The main objective of this study was to further improve diagnostic potential of convolutional neural networks (CNNs) in detection of lymph node metastasis in breast cancer patients by integrative augmentation of input images with multiple segmentation channels. For this retrospective study, we used the PatchCamelyon dataset, consisting of 327,680 histopathology images of lymph node sections from breast cancer. Images had labels for the presence or absence of metastatic tissue. In addition, we used four separate histopathology datasets with annotations for nucleus, mitosis, tubule, and epithelium to train four instances of U-net. Then our baseline model was trained with and without additional segmentation channels and their performances were compared. Integrated gradient was used to visualize model attribution. The model trained with concatenation/integration of original input plus four additional segmentation channels, which we refer to as ConcatNet, was superior (AUC 0.924) compared to baseline with or without augmentations (AUC 0.854; 0.884). Baseline model trained with one additional segmentation channel showed intermediate performance (AUC 0.870-0.895). ConcatNet had sensitivity of 82.0% and specificity of 87.8%, which was an improvement in performance over the baseline (sensitivity of 74.6%; specificity of 80.4%). Integrated gradients showed that models trained with additional segmentation channels had improved focus on particular areas of the image containing aberrant cells. Augmenting images with additional segmentation channels improved baseline model performance as well as its ability to focus on discrete areas of the image.

## 1. Introduction

Whether metastatic lesions are present in sentinel lymph nodes (SLN) is an important prognostic marker for early-stage breast cancer [1]. Large tumor size and perivascular invasion are associated with SLN involvement [2]. Therefore, the presence of metastatic tissue in SLN of breast cancer patients often represents a disseminated disease associated with poor prognosis and limited treatment options [3,4]. Since the status of SLN cannot be determined by clinical examination alone, SLN biopsies are routinely performed on early-stage breast cancer patients and are assessed by clinical pathologists for metastasis [1].

Accurate histopathological diagnosis empowers clinicians to recommend targeted treatment options specific for each patient [5]. Such histopathological diagnoses often occur in a time-limited setting during surgery, requiring a rapid classification of metastatic status, which greatly influences intraoperative decisions made whether to proceed with invasive treatment options or not [5,6]. For example, SLN-positive patients are recommended to receive axillary lymph node dissection, which is associated with significant permanent impairment [1]. However, detection procedures conducted by pathologists are often time consuming and subjective [5,7]. For example, metrics such as tumor cell percentage or quantification of fluorescent markers for estrogen receptor and/or HER-2 status are tasks that are often associated with inter-observer variability [8]. Furthermore, for the task of micro-metastases detection under simulated time constraints, pathologists have shown an underwhelming performance of 38% [3].

Whole-slide imaging systems have improved over the years, and are now capable of producing digitized, high-resolution, giga-pixel whole-slide images (WSI) of histopathology slides [9]. Using this technology, histopathological assessments can be done on a computer screen rather than using light microscopes. Digitization of workflow in pathology laboratories can reduce patient identification errors and save time for both pathologists and laboratory technicians [8]. Digitization of WSI has also enabled the development of automated computer-assisted diagnosis (CAD) platforms [9,10]. Automated computer-assisted diagnosis (CAD) has the potential to improve the speed and accuracy of histopathological diagnoses as well as reducing subjectivity [5,6,7,8,10].

Advancements in computer vision, most notably deep learning, has enabled researchers to extract more abstract features from large amounts of high-resolution medical images [6,11]. Therefore, high-resolution WSIs that contain complex features are suitable for application of deep learning strategies using convolutional neural networks (CNNs) [12]. The Cancer Metastases in Lymph Nodes Challenge 2016 (Camelyon16) found best algorithms to be performing significantly better than pathologists with time constraints and comparable to pathologists without time constraints [3]. Lymph Node Assistant (LYNA), an algorithm developed by Google AI Healthcare [5], managed to achieve 99.0% area under the curve in detection of micro- and macro-metastases from lymph node blocks [10]. Furthermore, pathologists with assistance from LYNA achieved 100% specificity and showed improved sensitivity over performance achieved by LYNA alone, which suggests the benefit of human intervention in CAD and room for improvement [10].

Weights previously trained on large-scale datasets such as ImageNet [13] can be used to initiate training of the model on a different task. Such strategy known as transfer learning have reportedly shown to facilitate faster convergence and better prediction performance for CNNs in digital pathology [7,14]. For example, Nishio et al. [15] have shown that VGG16 [16] with transfer learning performed better overall than same models trained without transfer learning. However, transfer learning does not guarantee better performance, because performance of models trained with the same architecture and pre-trained weights have been observed to differ greatly [4].

Data augmentation strategies, such as stain color normalization and morphological transformations of the input images, are often employed for digital histopathology image analyses, to improve model generalizability and robustness [12,17,18]. Algorithms such as WSI color standardizer (WSICS) [19] and Stain Normalization using Sparse AutoEncoders (StaNoSA) [17] demonstrated that data augmentation can improve performance of existing CAD systems for tasks such as necrosis quantification and nuclei detection, respectively. Therefore, we sought other data augmentation approaches to further improve performance of existing CAD models in histopathology.

Pathologists look for histological features such as nuclei, mitotic figures, tissue types, and multicellular structures such as tubules to make and justify their diagnoses. For example, pixel-wise detection of cytological features such as epithelial cell nuclei, epithelial cell cytoplasm, and the lumen were used for the higher-level tasks of gland segmentation and prediction of tumor grade on the Gleason grading scheme in prostate cancer [20,21]. Another study showed that local descriptors such as the distribution of cell nuclei was one of the most significant features used by a random forest model to detect metastasis from digital pathology images [22].Therefore, we investigated if we could further improve the performance of baseline CNN models by providing multiple segmentation channels of the input images with pixel-wise histological annotations of such features. Each of these segmentation channels can be extracted by U-net, a CNN model designed for semantic segmentation of biomedical images [23], which can then be integrated onto the original images depth-wise prior to input into the baseline model. We hypothesized that training CNN models with additional multiple segmentation channels will boost its performance over the baseline model. The specific aims of this project were: (1) train and evaluate a baseline CNN model for detecting breast cancer metastasis from digital histopathology images of lymph node sections using the PatchCamelyon (PCam) dataset [12]; (2) train four instances of a U-net model for semantic segmentation of histological features including the nucleus, mitotic figures, epithelium, and tubule using four independent datasets curated previously [24]; (3) train and evaluate a second instance of the baseline model with additional segmentation channels of images from the same test set to compare to the baseline model.

## 2. Results

### 2.1. Summary of Methods

**Figure 1:**
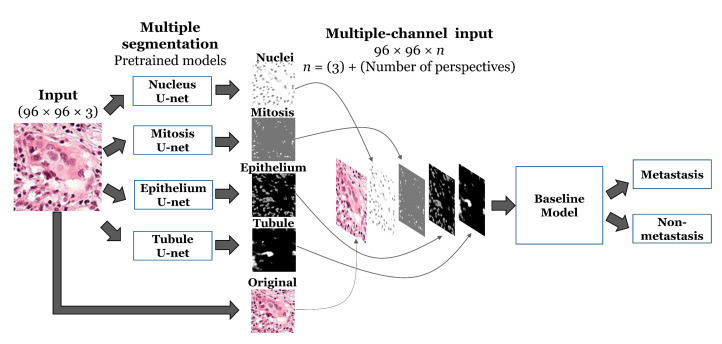
Visual representation of the proposed ConcatNet model.

We used PCam as our benchmark dataset to compare performance between models for detection of metastases from patches of lymph node sections [12]. A CNN model with a repeated series of a 3 × 3 depth-wise convolution, batch normalization, and max pooling layers was used as the baseline binary classifier, subsequently referred to as the ‘baseline’ model (Figure S1). To investigate how the same baseline model would perform if given data integrated with additional segmentation channels, we used U-net (Figure S2), a neural network architecture for biomedical image segmentation [23], along with four independent histology datasets [24] to generate semantic segmentation masks for four histological features: nucleus, mitotic figures, epithelium, and tubules (Figure S2). Therefore, for a given input, 4 U-nets, pre-trained for semantic segmentation of four histological features, extracted different perspectives of the input image, which were then concatenated to the original input image as additional channels depth-wise. We trained the baseline model with original input image integrated with segmentation masks for each histological feature separately, as well as altogether. Resulting integrated input image was given to the baseline model for the binary classification problem. We also compared the performance of our models against the baseline model trained with conventional data augmentation techniques, such as rotations, shifts, and flips. Details on the model schematic can be referred to Section 4.2 and Figure 1, which we subsequently refer to as ‘ConcatNet’.

### 2.2. Training and Validation of the Models

As shown in Table 1, each of the U-net models was trained to reach sufficient validation accuracies for segmenting the nucleus (96.55%), mitosis (95.44%), epithelium (82.92%), and tubule (84.64%), respectively. Figure S3 showed the records of training and validation accuracies for U-net models across different training epochs, which motivated us to select different epochs for each of the U-net models shown in Table 1.

The baseline CNN model was trained for 50 epochs reaching validation accuracy of 81.93% and lowest validation loss of 0.5657 when its training accuracy was 95.25% and training loss was 0.1247. We also trained the baseline CNN model with conventional data augmentations, which resulted in a slightly lower performance than the baseline without data augmentations.

VGG16 model with pre-trained weights from ImageNet dataset was trained as an example of a conventional transfer learning strategy. VGG16 converged at higher training accuracy of 99.75% but showed lower validation accuracy of 79.00%, characteristic of overfitting. Note that because a different preprocessing function was used for VGG16, its loss values should not be compared directly with other models.

In comparison, we trained the baseline model with input data integrated with additional semantic segmentations of various histological features, which is referred to as, ConcatNet. ConcatNet was also trained for 50 epochs and it reached validation accuracy of 86.23% and validation loss of 0.4357 when its training accuracy was 95.90% and training loss was 0.1082. Other models trained with one additional segmentation channel also converged to similar training accuracies and loss values (Table 2). As intended, the total number of parameters increased with number of additional segmentation channels; however, the number of trainable parameters were held relatively constant with increase of only 41 trainable parameters for each additional perspective. This increase occurred in the first depth-wise separable convolutional layer following the concatenation layer that increased the depth dimension of the input image for each additional perspective. This increase by 41 is due to the first depth-wise convolution layer consisting of two separate sets of convolutional filters. In other words, for input image of size 96 × 96 × *n*, where *n* is the number of color channels in the original image, plus each additional segmentation channel concatenated to the image: (1) *n* kernels of size 3 × 3 pixels, resulting in 9 additional trainable parameters; and (2) 32 kernels of size 1 × 1 × *n*, resulting in 32 additional trainable parameters.

### 2.3. Model Performance on the Test Set

Performance of all models on the test sets were visualized as confusion matrices as shown in Figure S4. The baseline model, performed well in identifying the negative samples with specificity of 80.4% but suffered from a relatively poor sensitivity of 74.6%, resulting in accuracy of 76.4% on the test set. In contrast, ConcatNet performed even better than baseline in identifying both negative (specificity 87.8%) and positive (sensitivity 82.0%) samples, resulting in accuracy of 84.1% on the same test set. Therefore, ConcatNet, which was trained with additional segmentation channels, consistently performed better on both validation and test set images than the baseline.

**Figure 2:**
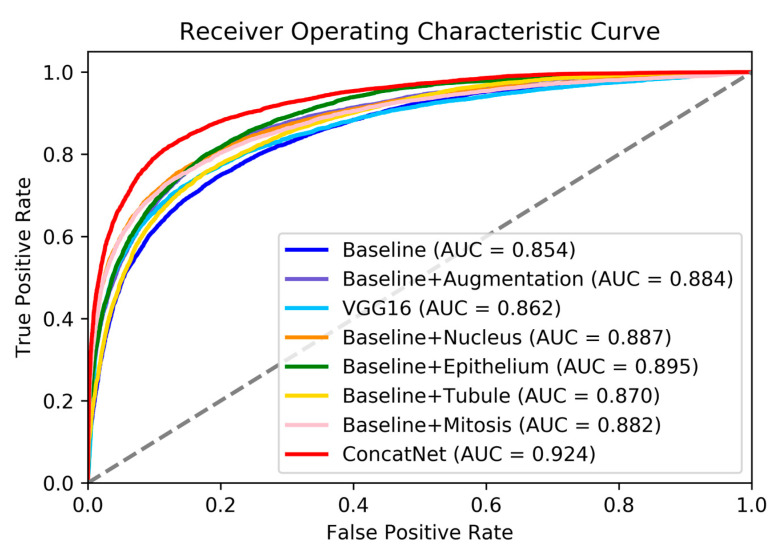
Receiver Operating Characteristic (ROC) curves for all models evaluated on the test set. Dotted line indicates AUC = 0.5.

We examined receiver operating characteristic (ROC) curves for all models we trained in this study, as shown in Figure 2. ConcatNet had the highest AUC of 0.924 for the test set, whereas the baseline model had the lowest AUC of 0.85. Other models that were trained with only one additional segmentation channel had AUC in between the baseline and ConcatNet (AUC 0.870–0.895), which was consistent with what was observed from confusion matrices. Baseline model trained with data augmentations showed similar performance to models with only one additional segmentation channel (AUC = 0.884). The numerical results of predictions on the test set are collectively shown in Table 3. By using the integrated gradients (IG) algorithm [25,26], we sought to examine how the models were interpreting the images; in other words, whether they were directing attention to those pixels containing metastatic cancer cells. As a baseline image for IG algorithm, we used a blank image filled with zero-pixel values. In the explanation of the original input image generated by IG algorithm, red pixels signify regions with positive attribution, where higher pixel intensity (moving away from baseline of 0) contributes to an increasing in the prediction score towards a positive label (containing metastatic cells). Similarly, blue pixels show regions with negative attribution, where higher pixel intensity contributes to a negative label (not containing any metastatic cells); or conversely, lower pixel intensity towards the baseline of 0 causes the prediction score to increase. White pixels seemingly do not contribute to the models’ prediction scores.

**Figure 3:**
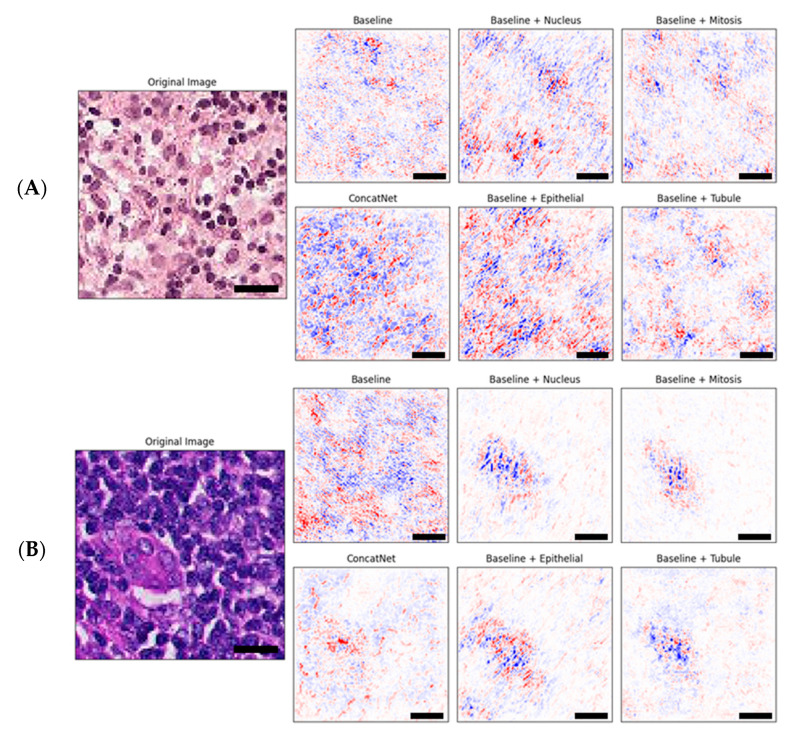
Example sets of explanations of model attribution for each of the baseline and the proposed models evaluated as compared to the original input image shown on the left. Red is positive attribution towards classification and blue is negative attribution towards classification. Scale bars (black) are shown for 10 microns (10 μm). Original (input) image for (**A**) shows a benign tissue section while original image for (**B**) contains malignant tumor cells.

The combination of both red and blue pixels indicates those regions of interest that are regarded as highly important for the model in assigning a class label to the entire image [27,28]. As a result, we observed that in the explanations by IG, there was a tendency for pixels containing cell nuclei to be associated with negative attribution (blue) because they were consistently represented by darker blue hematoxylin dye, whereas pixels containing the cytoplasm and cell junctions were associated with positive attribution (red) because they were consistently represented by lighter pink eosin dye. Figure 3A shows an example of this bias, which should be verified by clinical pathologists.

Figure 3B shows that training the model with one or more additional segmentation channels enabled the neural network to shift focus to those pixels more likely to contain malignant cells as compared to the baseline model which seemed to have a rather spread out focus that spanned across the entire image. Better focus, however, did not necessarily indicate better decisions made by the model. With regards to the input image in Figure 3B, only ConcatNet was able to assign a correct positive label to the image, whereas all other models including the baseline incorrectly assigned a negative label.

## 3. Discussion

Deep neural networks were inspired by the organization of the human visual cortex [29]. By designing a model which mimics the human brain, researchers were able to gain significant advances in various fields, notably in computer vision and CAD [5,6,30,31]. Likewise, the central motivation of this study was to modify a model to mimic how a pathologist sees a histology image and assess the model’s performance. In the eyes of a pathologist, histological features like cell nuclei, cell type, cell state, and multicellular structures are recognized naturally, which all contribute to the pathologist’s ability to recognize malignancy from a given histology image [21]. Objective and quantitative segmentation of histologic primitives such as the nuclei and glandular structures is one of the major interests of digital pathology [11]. Accordingly, we extracted multiple segmentation channels that captured such histological features, which were used to augment input images during the training phase. As previously demonstrated by the whole-slide image color standardizer (WSICS) algorithm, which reduced the effects of stain variations and further improve performance of a CAD system by incorporating spatial information, we incorporated the spatial information of histological structures to improve our model’s classification performance [19].

For our problem of detecting metastatic cells from digital histopathology images of sentinel lymph node sections extracted from breast cancer patients, we observed improvements in both sensitivity and specificity when the models were provided with one or more additional segmentation channels. Deep neural networks and features generated by these models have been criticized for their lack of interpretability [11]. However, we also showed that through the IG algorithm [25] that the models trained with additional segmentation channels were able to establish regions of interest containing malignant-looking cells or structures when the baseline model could not.

Our findings suggest that even for models of CAD with considerably high predictive performance, their performance can be further improved by augmenting input images with multiple additional segmentation channels. Diagnostic errors are expensive both for the patient and the healthcare system because false positive results can lead to unnecessary calls for additional diagnostic tests or treatments on a healthy individual, and false negative results can lead to a lack of care for patients who need early medical intervention [32,33]. Furthermore, both types of errors can lead to potential litigations. Therefore, it is important to consider our method of data augmentation to further improve the performance of existing CAD tools and those in development. However, it should be noted that although the IG algorithm was able to visualize the differences in feature attribution between models, we still do not have a clear understanding as to why some models have focused appropriately on regions containing malignancy and yet made incorrect decisions on some of the images. Nonetheless, proper focus and extraction of regions of interest can potentially relieve the burden of pathologists, who serve majority of their time scanning benign areas without malignancy [21]. Moreover, the ability of automated CAD tools to speedily and objectively quantify histopathological features such as tumor cell percentage and disease grade is much needed [8].

Many of our predecessors in digital histopathologic image analysis have used transfer learning techniques, mostly by using weights from CNN architectures pre-trained on large generalized image datasets such as ImageNet [13], to reduce training time and to benefit from potential performance benefits [7,14,15,34]. Although there was a significant reduction in training time, the performance results were highly variable, even with the same pre-trained CNN architectures [4]. In our study, we observed that VGG16 with transfer learning performed better than the baseline, albeit with substantially higher number of parameters. Our approach to augment the training phase of CNN models can also be seen as a method of transfer learning, albeit different from our predecessors in that (1) we transferred knowledge gained from the same type of images, specifically from histopathology; and (2) rather than transferring only the weights, we used entire pre-trained networks in parallel to extract new segmentation channels from the same input image [34]. These two key differences potentially contributed to the improvements in performance benefits that were observed in this study, including convergence at lower loss value and increased generalizability to unseen data, with little additional computational cost to the classifier models.

However, a major limitation of this study was that the annotated histology images used to train the U-nets were not from the same tissues. For example, the nuclei and tubule segmentation datasets were images from colorectal cancer patients [35] whereas the epithelium and mitosis segmentation datasets were images from breast cancer patients [24]. Furthermore, our main benchmark dataset, PCam, consisted of images from sentinel lymph node sections [3,12]. Training the U-nets and the subsequent baseline model with a single dataset with multiple annotations for nuclei, mitotic figures, multicellular structures, and other histological features has potential to improve model performance even further.

## 4. Materials and Methods

This project was retrospective; we used histopathology datasets that were publicly available online to train the CNN models. Patient information or clinical features were neither necessary nor used for this project.

### 4.1. Datasets

For the main benchmarking dataset, we used the PatchCamelyon (PCam) dataset of digital histopathology images of lymph node sections from breast cancer patients [3]. PCam consists of 327,680 images divided into training (80%: 262,144), validation (10%: 32,768), and test (10%: 32,768) sets. The separation strategy was used because each of the data sets carried sufficient number of samples to train, validate, and test models in a robust manner as done by others as well [3,12,31]; hence, we did not employ k-fold cross validation approaches. Images 96 × 96 pixels in size with 3 channels representing the RGB (red, green, blue) color, are non-duplicated segments of the Camelyon16 dataset at 40× apparent magnification. Images from PCam are associated with a binary label for the presence (1) or absence (0) of metastatic breast cancer tissue in the center 32 × 32-pixel area of the image. All subsets of the PCam dataset consisted of an equal proportion of positive (1) and negative (0) samples; i.e., training set contained 131,072 images for each class (positive and negative), whereas the validation and test sets each contained 16,384 images in each class.

Datasets used to train our U-net models for segmentation/detection of mitotic figures, epithelium and tubule from digital histology images were obtained online from a previous study, accessible at http://www.andrewjanowczyk.com/deep-learning/ [24]. Dataset for mitosis detection contains 311 images of size 2000 × 2000 at 40× selected from 12 breast cancer patients and the ratio of positive to negative pixels was around 1:6664. Dataset for epithelium segmentation contains 42 images of size 1000 × 1000 at 20× selected from estrogen receptor-positive breast cancer patients and the ratio of positive to negative pixels was around 1:2. Dataset for tubule segmentation contains 85 images of size 775 × 522 at 40× from colorectal cancer patients and the ratio of positive to negative pixels was 1:1.13. Each image in the mitosis, epithelium and tubule datasets were associated with ground truth mask images with pixel-wise annotations for mitosis, epithelium, and tubule respectively.

Our cell nuclei dataset, CRCHistoPhenotypes, consisting of 100 digitized H&E (Hematoxylin and eosin) stained histopathology images of colorectal adenocarcinomas, was downloaded from University of Warwick website [35]. Each image is a non-overlapping patch of size 500 × 500 pixels cropped from 10 WSIs at 20× optical magnification. Center pixel coordinates for a total of 29,756 cell nuclei were annotated and validated by a pathologist. In its original form this dataset suffers from class imbalance because cell nuclei annotations were significantly outnumbered by non-nuclei pixels (1:840). We resolved this problem by training multiple instances of U-net on mask images with annotations expanded by *n* pixels (i.e., morphological dilation of label masks images). We selected U-net trained with nucleus annotations expanded by 5 pixels as the model predicted blank images when the label pixels were expanded by only 0–3 pixels, and it produced results most representative of actual nuclear area depicted in the original input images with the highest validation accuracy. After dilation of label masks, the ratio of nuclei to non-nuclei pixels was around 1:6.

We pre-processed each of the datasets to match the resolution of our benchmark dataset, PCam, with apparent magnification of 40× and image size 96 × 96 × 3.

### 4.2. Deep Learning Models

We implemented all of our deep learning models on Keras API (version 2.4.0) with TensorFlow (version 2.3.0) backend using the Python programming language (version 3.6.9).

For the semantic segmentation of histological features, we implemented the U-net architecture obtained from [36] as shown in Figure S2. We trained four instances of the U-net model on the four different datasets described above to segment for cell nuclei, mitotic figures, epithelium, and tubules. In order to ensure portability of our model for subsequent analyses on the PCam dataset, we pre-processed each of the four feature datasets by cropping and rescaling to image size 96 × 96 and apparent magnification of 40×, to match the resolution of the PCam dataset. Each U-net model was trained with batch size of 1, the Adam optimizer with Nesterov momentum [37] with initial learning rate of 1 × 10^−5^, and binary cross-entropy loss function. However, due to the highly imbalanced nature of the ‘mitosis’ dataset, we used weighted binary cross-entropy loss function to train the U-net model for this dataset. Accuracy (ACC) and dice similarity coefficient (DSC) [38] were used as metrics for the segmentation task, with accuracy defined as the percentage of pixels that are correctly classified (pixel accuracy) and dice coefficient computed according to the following formula:

(1) $$DSC=\frac{2\left|X\cap^{\;}Y\right|}{\left|X\right|+\left|Y\right|}$$

where *X* and *Y* represent two samples—in our case the predicted segmentation mask and the actual mask.

The level of difficulty for each segmentation tasks varied, so we trained the U-nets with different number of epochs accordingly by observing the trends during the training phase and selected the number of epochs where each of the U-net models trained to reach sufficient validation metrics; refer to Figure S2.

For the baseline binary classifier, we adapted a simple sequential CNN model originally proposed for detecting invasive ductal carcinoma from breast cancer histopathology images as baseline model, with no additional segmentation channels [39]. The architecture of the baseline model consists of several repeats of 3 × 3 depth-wise convolutional layers followed by batch normalization and max pooling layers (Figure S1). The final layer of the baseline model was modified to contain only one node with sigmoid activation function, which was interpreted as the predicted likelihood that the input image contains metastatic cells. The baseline model was trained with batch size 32, the Adam optimizer with learning rate of 1 × 10^−4^, and binary cross entropy loss function for 50 epochs without any further data augmentations on the PCam training dataset. We also trained the baseline model with random data augmentations such as width shifts [−0.25, +0.25) height shifts [−0.25, +0.25), rotations (0–180°), horizontal flips, and vertical flips, otherwise using the same hyperparameters.

For the VGG16 model with transfer learning, we used VGG16 model [16] in Keras loaded with weights from training on the ImageNet dataset for the 1000-class classification task [13]. We froze the convolutional layers but made the top fully connected layers trainable as well as changing the classifier layer to a sigmoid activation function with one node since our task was for binary classification. We used the ‘*preprocess_input*’ function to preprocess input images from RGB to BGR, then zero-centering all channels with respect to the ImageNet dataset without scaling. Table S1 summarized model hyperparameters used to train all models in this paper.

To provide our baseline model with additional segmentation channels, we included the outputs from four pre-trained U-net models as inputs. Specifically, before the first convolutional layer we added a layer to concatenate outputs from U-net models as four additional channels along with the original image; hence, we referred to the new CNN architecture as ConcatNet. A visual representation of our proposed model is shown in Figure 1. ConcatNet was trained with the same hyperparameters as compared to the baseline on the PCam training dataset. We also trained four additional instances of the baseline model with only one additional segmentation channel and benchmarked their performance.

### 4.3. Performance Benchmarking

To keep track of the model during its training phase, we used the validation set from PCam that does not contain images from the training set to monitor its performance. The 32,768 images of the PCam test set which were not shown to the models previously during training were used to benchmark model performance. Given an input image from the test set, models predicted the likelihood of whether each input image contained metastatic tumor cells or not. Confusion matrices and receiver operating characteristic (ROC) curve were used as measures of model performance. For calculation of model metrics of the test set in Table 3, we computed the Youden Index (J) [40] using predictions from the models to determine the optimal cut-point for prediction outputs with the following formula:

(2) J=sensitivity+specificity−1

Integrated gradients (IG) algorithm [25] was used to visualize differences in interpretations of the same input image between models.

## 5. Conclusions

In summary, we demonstrated that improvements were made in both sensitivity and specificity when deep learning models were trained with additional segmentation channels of input images. IG analysis suggested that these additional segmentation channels help the models to orient their attention to specific regions of the image containing malignancies, although we found examples where better focus did not necessarily lead to correct classification. However, further analyses should be repeated using larger datasets with better resolutions and deeper models in the future to investigate if our results can be replicated under those circumstances. Interpretation of deep learning models still remains a challenge and presents room for improvements.

Furthermore, the feature segmentation pipeline using U-net can be extended to segment other, more complex histological features such as different tumor tissues, inflammation, and necrosis, among many others. We demonstrate that data augmentation with prior extracted features have potential to further improve the performance of CAD tools in digital histopathology and other tasks in medical image analyses, in which even small improvements in performances has significant implications for the patient’s clinical outcomes.

## Figures and Tables

**Table 1:** Results on the training of U-net models.

Feature	Epochs	Training Accuracy	Train Dice Coefficient	Valid Accuracy	Valid Dice Coefficient
Nucleus	50	0.9424	0.9751	0.9655	0.9845
Mitosis	1000	0.7734	0.8004	0.9544	0.8004
Epithelial	300	0.7951	0.9114	0.8291	0.8532
Tubule	1000	0.9598	0.9455	0.8464	0.9399

**Table 2:** Results on the training and validation sets for models evaluated.

Model	Number of Parameters (Trainable)	Number of Epochs Trained	Training Accuracy	Training Loss	Validation Accuracy	Validation Loss
Baseline	4,772,220	50	95.25%	0.1247	81.93%	0.5657
Baseline + Augmentation	4,772,220	50	93.82%	0.1640	82.13%	0.6210
VGG16	35,663,873	50	99.75%	0.0081	79.00%	2.9023
Baseline + Nucleus U-net	4,772,261	50	95.85%	0.1923	83.36%	0.5808
Baseline + Mitosis U-net	4,772,261	50	95.28%	0.1236	83.97%	0.4597
Baseline + Epithelium U-net	4,772,261	50	95.23%	0.1261	85.07%	0.4045
Baseline + Tubule U-net	4,772,261	50	96.02%	0.1048	82.93%	0.6176
ConcatNet (Baseline + all U-nets)	4,772,384	50	95.90%	0.1082	86.23%	0.4357

**Table 3:** Results on the test set for models evaluated.

Model	Sensitivity	Specificity	Accuracy	AUC
Baseline	74.6%	80.4%	76.4%	0.854
Baseline + Augmentation	80.2%	81.4%	78.8%	0.884
VGG16	75.3%	82.6%	76.5%	0.862
Baseline + Nucleus U-net	75.4%	86.6%	77.7%	0.887
Baseline + Mitosis U-net	74.2%	86.9%	79.9%	0.882
Baseline + Epithelium U-net	80.0%	82.3%	79.3%	0.895
Baseline + Tubule U-net	76.1%	86.9%	76.1%	0.870
ConcatNet (+all U-nets)	82.0%	87.8%	84.1%	0.924

## Supplementary Materials

The following are available online at https://www.mdpi.com/2072-6694/12/10/2934/s1, Figure S1: Architecture of baseline model used in this study, Figure S2: U-net structure for image segmentation, Figure S3: Record of training and validation accuracies across epochs for U-nets trained for semantic segmentation, Figure S4: Confusion matrices for models evaluated on the test set, Table S1: Model hyperparameters.
