# Supplementary material for: Integrative Data Augmentation with U-Net Segmentation Masks Improves Detection of Lymph Node Metastases in Breast Cancer Patients
Source: Cancers (Basel). 2020 Oct 12;12(10):2934. doi: 10.3390/cancers12102934 (PMC7601653; doi:10.3390/cancers12102934)
Supplement: Supplementary file 1 [file cancers-12-02934-s001.pdf]

Supplementary Material: Integrative Data Augmentation with U-net Segmentation Masks Improves Detection of Lymph Node Metastases in Breast Cancer Patients

Yong Won Jin, Shuo Jia, Ahmed Bilal Ashraf and Pingzhao Hu

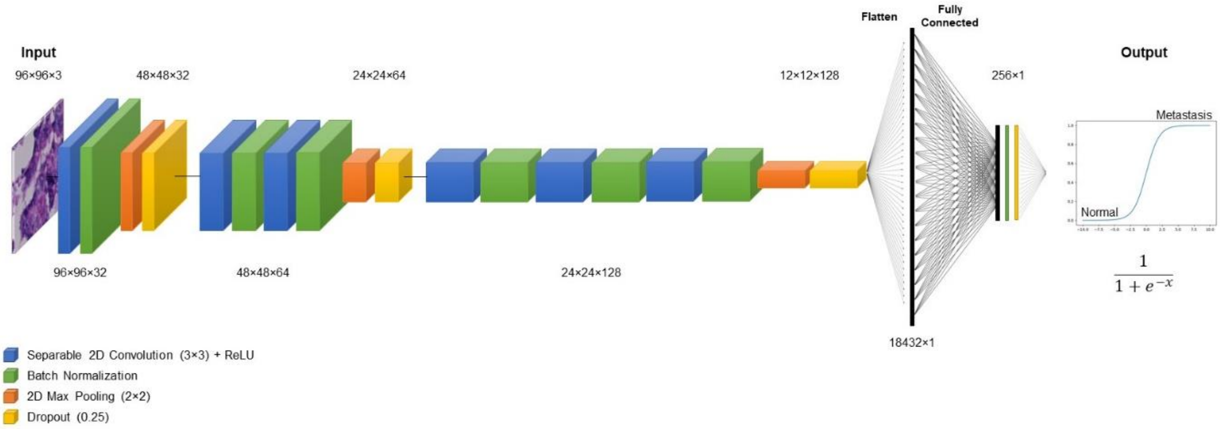

Figure S1. Architecture of the baseline model used in this study. It resembles the VGG16 network but instead of traditional convolutional filters, it takes advantage of depth-wise convolutional filter layers for reduced number of parameters. We further modified the input layer as well as filter dimensions in the last few layers to accommodate the architecture to our problem.

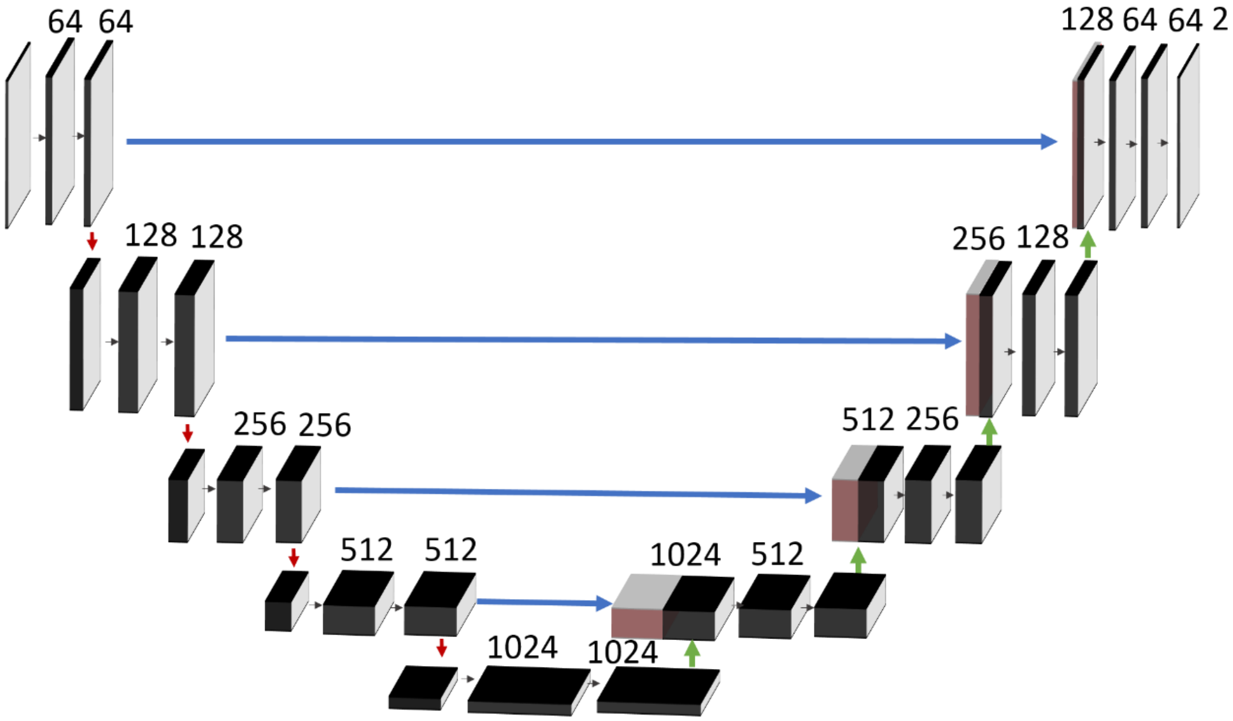

Figure S2. U-net structure for image segmentation. We applied U-net on histology images to extract multiple perspectives containing different histological features. U-net can be trained efficiently for image segmentation tasks even with few training images. Black boxes are 3 × 3 convolutional layers with ReLU, while blue arrows are copy and concatenate procedures, red arrows are 2 × 2 max pooling layers and green arrows are 2 × 2 up-convolutional layers.

A

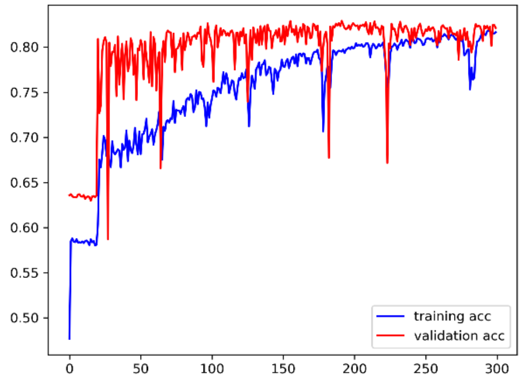

B

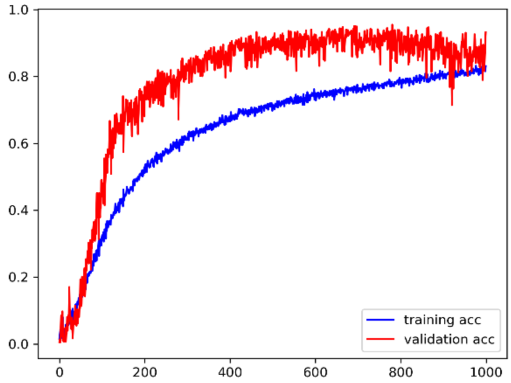

C

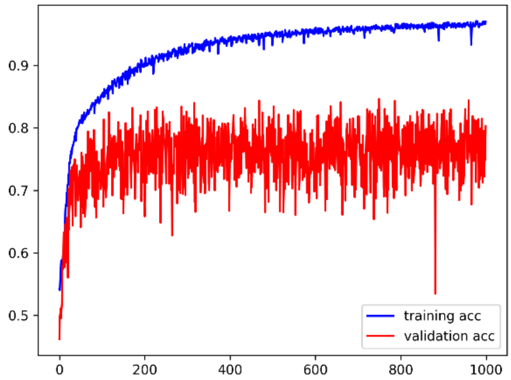

Figure S3. Record of training and validation accuracies across epochs for U-nets trained for semantic segmentation of (**A**) epithelium; (**B**) mitosis; and (**C**) tubule. Metrics were not explicitly recorded for nucleus U-net and was therefore, omitted.

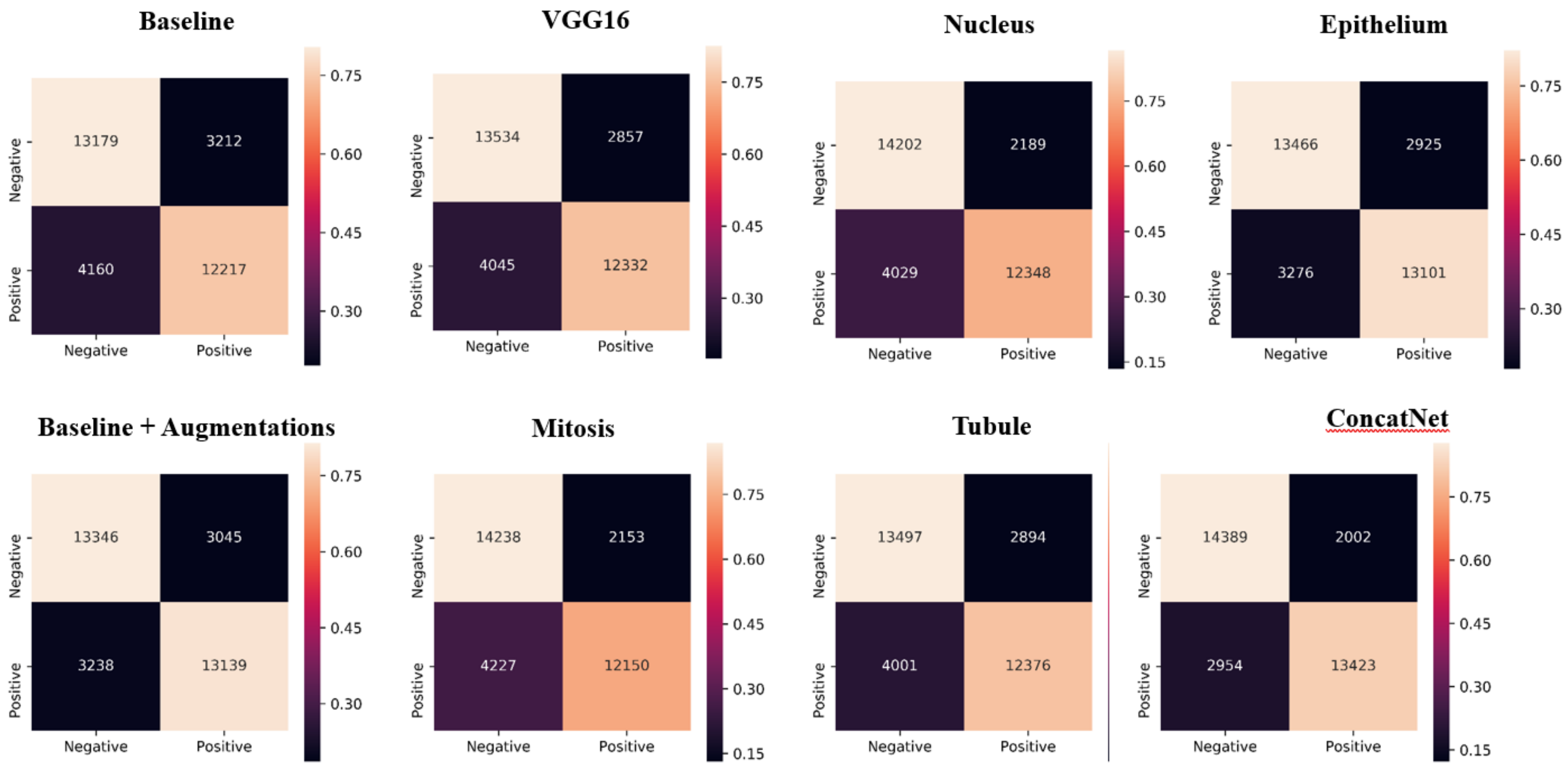

Figure S4. Confusion matrices for models evaluated on the test set. Y-axis represents actual labels (lymph node positive vs. negative), and X-axis represents predicted labels. As the scales indicate, lighter color indicates rate closer to 1 and darker color indicates rate closer to 0.

Table S1. Model hyperparameters.

Model	Number of parameters (trainable)	Epoch	Train Steps	Valid Steps	Batch Size	Loss Function	Optimizer	Learn Rate
U-net (Nucleus)	31,032,837	50	90	10	1	Binary cross-entropy	NAdam	1×10^{-5}
U-net (Mitosis)	31,032,837	1000	252	27	1	Weighted binary cross-entropy	NAdam	1×10^{-5}
U-net (Epithelium)	31,032,837	300	38	4	1	Binary cross-entropy	NAdam	1×10^{-5}
U-net (Tubule)	31,032,837	1000	77	8	1	Binary cross-entropy	NAdam	1×10^{-5}
Baseline	4,772,220	50	8192	1024	32	Binary cross-entropy	Adam	1×10^{-4}
VGG16	35,663,873	50	8192	1024	32	Binary cross-entropy	Adam	1×10^{-4}
U-net + Baseline	4,772,261	50	8192	1024	32	Binary cross-entropy	Adam	1×10^{-4}
ConcatNet	4,772,384	50	8192	1024	32	Binary cross-entropy	Adam	1×10^{-4}
